# Association between Post-Hospitalization Psychological Distress, Exercise Capacity, Physical Function and Health Status in COVID-19 Survivors

**DOI:** 10.3390/healthcare12050577

**Published:** 2024-03-01

**Authors:** Clara D’Ors-Vilardebó, Maria Àngels Cebrià i Iranzo, Carola González-King-Garibotti, María Isabel Vázquez-Arce, Andrés Calvache-Mateo, Laura López-López, Marie Carmen Valenza

**Affiliations:** 1 Physical Medicine and Rehabilitation Service, La Fe Hospital in Valencia, La Fe Health Research Institute (IISLAFE), 46026 Valencia, Spain; 2 Physiotherapy Department, University of Valencia, 46026 Valencia, Spain; 3 Physiotherapy Department, Faculty of Health Sciences, University of Granada, Av. De la Ilustración, 60, 18016 Granada, Spain

**Keywords:** COVID-19, exercise capacity, health status, physical functional performance, psychological distress

## Abstract

This study aims to determine whether post-hospitalization psychological distress is associated with exercise capacity, physical function and health status in COVID-19 survivors. In this observational study, hospitalized COVID patients were included and divided into two groups according to the mental component summary subscale of the 12-item Short-Form Health Survey. Patients with a score ≤ 45 were included in the psychological distress group, and patients with a score > 45 were included in the non-psychological distress group. The main variables were exercise capacity, physical function, and health status. Patients were evaluated at discharge, 3 months, and at 6 months follow-up. Finally, a total of 60 patients were included in the study. Significant differences were found in exercise capacity, physical function, and health status (*p* < 0.05), with worse results in the group with psychological distress at discharge and 3 months follow-up. At 6 months after discharge, COVID patients with psychological distress exhibited worse results in exercise capacity, physical function, and health status, being significant exercise capacity and physical function (*p* < 0.05). It can be concluded that COVID patients with psychological distress at hospital discharge reported worse exercise capacity, physical function and health status at hospital discharge, 3 months and 6 months follow-up.

## 1. Introduction

The Coronavirus disease 2019 (COVID-19) is an infection caused by Severe Acute Respiratory Syndrome Coronavirus 2 that leads to pneumonia [1]. It was first identified in China in late 2019 and rapidly spread to the rest of the world, becoming a global health emergency [2]. As of 4 August 2023, there were more than 769 million confirmed cases of COVID-19 worldwide [3]. 

The symptoms of COVID-19 encompass a spectrum of clinical presentations, with fever, dry cough, and shortness of breath being the predominant manifestations [4,5]. Numerous studies have underscored these symptoms as the most prevalent in individuals infected with the SARS-CoV-2 virus. Additionally, a diverse array of clinical features has been observed, including fatigue, myalgia, loss of taste and smell, sore throat, and headache [6]. It is essential to emphasize that the clinical presentation of COVID-19 can vary significantly, ranging from asymptomatic instances to severe forms requiring hospitalization. This underscores the complexity and diversity of symptoms associated with the disease, highlighting the importance of a thorough comprehension of its clinical manifestations [4,5,6]. The symptoms mentioned above have led to a surge in hospitalization, many requiring prolonged intensive care unit stays and mechanical ventilation [7].

Due to the serious nature of the COVID-19 global health crisis, a significant proportion of patients studied experienced stress, post-traumatic symptoms and psychological distress [8]. Psychological distress is defined as an unpleasant emotional experience caused by a variety of factors, which can manifest as tension, fear, anxiety, psychological instability and even serious psychological disorders, such as depression [9]. Spencer-Segal et al. (2021) [10] found higher levels of anxiety, post-traumatic stress and loneliness after discharge in patients hospitalized with COVID-19 compared to those hospitalized without COVID-19, independent of pre-existing medical and psychiatric conditions or illness severity. Mazza et al. (2020) [11] found prevalence rates of around 50% for psychological morbidities such as depression or anxiety in COVID-19 survivors, caused by several factors including social isolation imposed during hospitalization. Other related factors were the unexpectedness of the illness, loss of control, sense of powerlessness and strong negative feelings of fear, guilt and helplessness. Stigma is also an important issue among patients with COVID-19 as it may negatively affect their return to daily life in the community [8,12]. 

Previous studies have related psychological distress to a poorer health status, loss of functionality, and disturbed sleep quality in other pathologies [13,14,15]. 

Numerous scientific investigations have explored the complex interplay between psychological stress and diverse factors during hospital stays, revealing the significant influence of mental wellness on patients’ overall health outcomes. Research conducted by Smith et al. (2019) [16] explored the correlation between psychological stress levels and the immune response in hospitalized individuals. The study found a significant association between heightened stress levels and compromised immune function, highlighting the need for holistic healthcare approaches that address not only the physical, but also the mental aspects of patient well-being during hospital stays.

In addition to immune function, studies such as the one conducted by Johnson et al. (2020) [17] have investigated the relationship between psychological stress and patient adherence to prescribed medical treatments during hospitalization. This research revealed that elevated stress levels were associated with lower adherence rates to medication regimens and therapeutic interventions. These findings underscore the importance of incorporating psychological support mechanisms within hospital settings to enhance patient compliance and improve overall health outcomes. Collectively, these studies contribute valuable insights into the multifaceted nature of the relationship between psychological stress and various health-related variables, emphasizing the need for comprehensive healthcare strategies that address both the physical and mental aspects of patient care during hospitalization.

Nevertheless, little research has been done about the influence of psychological distress on exercise capacity, physical function and health status in COVID-19 survivors. Hence, the objective of this study was to analyze the association between post-hospitalization psychological distress and exercise capacity, physical function, and health status in COVID-19 survivors. To achieve that aim, a longitudinal observational study was carried out, including patients hospitalized with a diagnosis of SARS-CoV-2. They were divided into two groups according to the presence of psychological distress at hospital discharge, which was evaluated using the 12-item Short Form Health Survey. In addition to the anthropometric and sociodemographic data, exercise capacity, physical function, and health status were assessed. Patients were followed at three and six months after hospitalization to observe the progress of the variables studied in both groups. In this way, an association between psychosocial distress and these variables in the medium and long term can be observed. This information will contribute to the growing body of research on the long-term effects of COVID-19, offering valuable insights for both clinical and research communities.

## 2. Methods

### 2.1. Research Hypothesis

It is expected that those who experienced higher psychological distress post-hospitalization will have reduced exercise capacity, diminished physical function, and poorer health status compared to those who experienced lower levels of psychological distress.

This research hypothesis is based on previous literature research that has revealed the correlation between psychological distress with other health aspects such as health status and functionality in hospitalized patients with other pathologies [13,14,15].

### 2.2. Design

This is a longitudinal observational prospective cohort study with a 6-month follow-up conducted at a university hospital from March 2021 to January 2022. This study was approved by the Hospital Ethics Committee (REF 2021-004-1), was conducted by the amended Declaration of Helsinki, and was written according to the STROBE checklist.

### 2.3. Participants

The main inclusion criteria were as follows: (1) hospitalization with a diagnosis of SARS-CoV-2, (2) age over 18 years, (3) signed the informed consent. Patients were excluded if they had cognitive impairment, or orthopedic, or neurological pathologies that can make it difficult to perform the tests.

### 2.4. Grouping

Participants were divided into two groups according to the presence of psychological distress at hospital discharge, assessed by the 12-item Short Form Health Survey (SF-12) [18,19]. The SF-12 is a questionnaire designed to assess general self-rated health, physical and psychological symptoms, and limitations in everyday activity due to physical and mental health over the previous 4 weeks. It consists of 12 items that address various dimensions, including general health, physical functioning, role limitations due to physical and emotional health, mental health, social functioning, and bodily pain. By condensing the SF-36, the SF-12 maintains its reliability and validity, making it a practical tool for assessing health outcomes in diverse populations across both clinical and research settings [18]. The SF-12 generates two summary scores: the Physical Component Summary (PCS) and the Mental Component Summary (MCS), providing a snapshot of an individual’s overall physical and mental well-being, respectively. These scores are derived through a weighted combination of the 12 items, offering a comprehensive yet efficient assessment of health-related quality of life [20]. The scores ranged from 0 to 100 with higher scores indicating greater general health. The cut-off score was defined as an MCS-12 of 45 according to the results of previous studies [21]. Patients with a score ≤ 45 were included in the psychological distress group, and patients with a score > 45 were included in the non-psychological distress group.

### 2.5. Outcomes

Anthropometric and sociodemographic data were collected from medical history such as age, body mass index (BMI), length of hospital stay, length of intensive care unit (ICU) stay, tabaquism, and comorbidities.

The main outcome measures were exercise capacity, physical function, and health status. Patients were evaluated at hospital discharge, three, and six months after hospitalization.

#### 2.5.1. Exercise Capacity

Exercise capacity was evaluated by the 6 min walking test (6MWT) [22]. The 6MWT is a submaximal exercise test in which participants walk along a 30 m corridor. Participants were instructed to walk at their own pace but to cover as much ground as possible in 6 min. At the end of the test, perceived exertion was assessed by the Modified Borg scale (0 no dyspnea to 10 maximum dyspnea) [23].

The six-minute walk test (6MWT) is a widely utilized clinical assessment to evaluate functional exercise capacity in various populations, including individuals with respiratory and cardiovascular conditions [24]. In addition, it is particularly valuable in assessing the impact of chronic diseases on daily physical activities and has been endorsed as a reliable tool for assessing exercise tolerance in diverse clinical settings [25].

The reliability of the Six-Minute Walk Test (6MWT) has been established in various populations and clinical settings, making it a widely used and accepted measure of functional exercise capacity. Reliability refers to the consistency and reproducibility of test results under similar conditions. Numerous studies have reported good test–retest reliability for the 6MWT in different populations, including individuals with respiratory diseases, cardiovascular conditions, and other health-related issues [26].

For instance, a study by Enright et al. (2003) [27] demonstrated that the 6MWT has excellent test–retest reliability in healthy individuals, with an intraclass correlation coefficient (ICC) of 0.89. Additionally, in patients with chronic obstructive pulmonary disease (COPD), the 6MWT has shown good reliability, with ICCs ranging from 0.91 to 0.95 [27,28]. The test’s reliability has also been investigated in heart failure patients, showing consistent and reproducible results [29]. 

These findings collectively support the notion that the 6MWT is a reliable tool for assessing functional exercise capacity across various populations, making it valuable for clinical and research purposes.

#### 2.5.2. Physical Function

Quadricep strength was evaluated with a portable hand-held dynamometer (Lafayette Manual Muscle Testing System, model 01163, Lafayette, IN, USA). The patient has to be seated with his/her knees and hips flexed at 90°. The evaluator has to apply a resistance to the anterior tibia during 5 s of maximal muscle contraction. The test was repeated 3 times on each leg with a minute rest between measurements. The highest value in pounds was selected for the analysis [30]. 

The reliability of measuring quadricep strength using a portable hand-held dynamometer has been a subject of investigation in various studies across different populations. Reliability in this context refers to the consistency and reproducibility of measurements obtained with the dynamometer. Several studies have reported good-to-excellent reliability for assessing quadricep strength using handheld dynamometry. A study by Bohannon and Andrews (1987) [31] found high intrarater reliability (ICC = 0.98) and interrater reliability (ICC = 0.99) when measuring isometric knee extension strength in healthy adults. Another study by Mentiplay et al. (2015) [32] investigated the reliability of hand-held dynamometry in assessing isometric quadriceps strength in individuals with knee osteoarthritis, showing good intrarater reliability (ICC = 0.91 to 0.96) and interrater reliability (ICC = 0.87 to 0.93). These findings suggest that hand-held dynamometry is a reliable method for assessing quadricep strength in both healthy and clinical populations.

#### 2.5.3. Health Status

Health status was assessed by a handgrip dynamometry Jamar^®^ Smart Hand Dynamometer from Patterson Medical (TEC-60: Lafayette Manual Muscle Testing System, model 01163, Lafayette, IN, USA; Productos Técnicos, EE.UU) that was individually adjusted for the size of the subject’s handgrip. Three measurements were made on each hand and the peak force was recorded [33,34].

Handgrip dynamometry is a reliable and widely utilized method for assessing health status, particularly muscle strength and overall physical function. This non-invasive test involves measuring the maximum force a person can generate by squeezing a hand-held dynamometer. Handgrip strength has been recognized as a valuable indicator of overall muscular strength and is associated with a variety of health outcomes, including mortality, morbidity, and functional decline in diverse populations [35]. Several studies have demonstrated the significance of handgrip strength as a predictor of various health-related events, such as cardiovascular events, disability, and frailty [36]. Given its simplicity, portability, and ability to reflect broader aspects of health, handgrip dynamometry has become an integral component of clinical assessments and research investigations aimed at understanding and monitoring individuals’ health status.

### 2.6. Statistical Analysis

The sample size was calculated by G Power 3.1.9.2. based on an unpublished pilot study carried out including seven subjects, with an effect size of 0.75, and a statistical power of 90%. The sample size calculation was 64 participants (32 per group). Nevertheless, we recruited 36 participants per group to allow for a dropout rate of 10% [37].

To analyze the data obtained, SPSS (Statistical Package for Social Sciences) version 23.0 statistical package was used. To describe sample baseline characteristics, descriptive statistics (i.e., mean ± standard deviation) were carried out. Additionally, the data normality was assessed by the Kolmogorov–Smirnov test. Finally, a two (psychological distress COVID patients vs. no psychological distress COVID patients) × three (discharge, 3 months, and 6 months follow-up) analysis of variance was performed. 

## 3. Results

**Figure 1:**
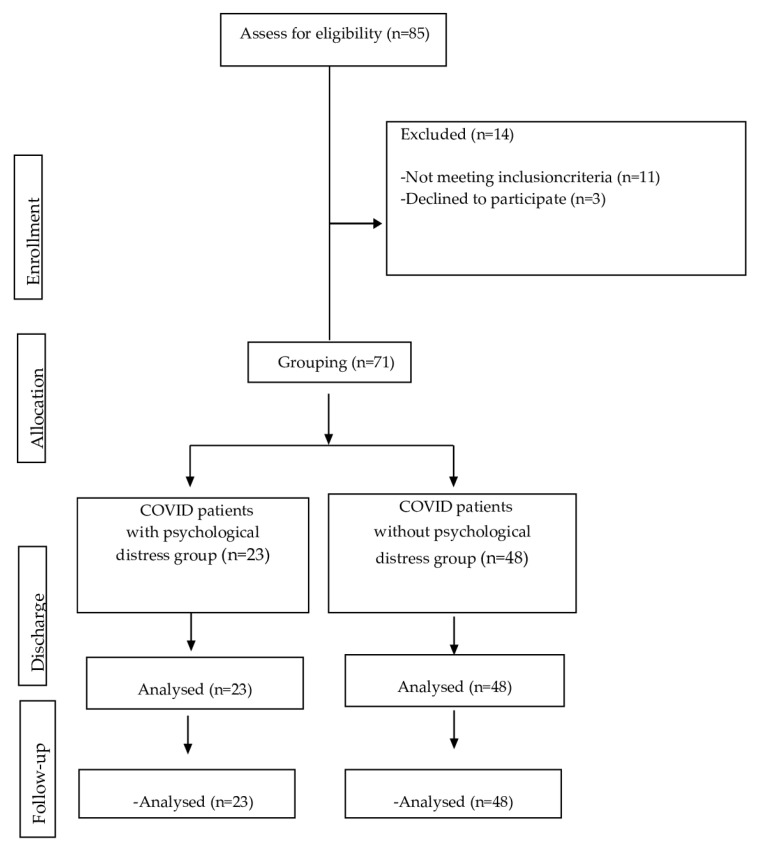
CONSORT flow diagram.

Of the 85 patients eligible for inclusion in the study, 14 were excluded; 11 did not meet the inclusion criteria and 3 declined to participate in the study. A total of 71 patients were grouped based on the presence of psychological distress. Finally, 23 patients were included in the COVID patients with psychological distress group, and 48 patients were included in the COVID patients without psychological distress group (see Figure 1).

The sociodemographic variables of the sample at hospital discharge are presented in Table 1.

As seen in Table 1, the mean age of COVID patients with psychological distress is 56.87 years, while those without distress have a slightly higher mean age of 61.63 years. Nevertheless, there is no statistically significant difference in the ages of the two groups (*p* = 0.126).

On the other hand, a statistically significant difference is observed in the distribution of sex. In the group with psychological distress, 78.26% are men, whereas in the group without psychological distress, the percentage is higher, at 95.83% (*p* = 0.020). Furthermore, there is a significant difference in the BMI between the two groups. COVID patients with psychological distress have a lower BMI (27.10 kg/m^2^) compared to those without distress (30.93 kg/m^2^) (*p* = 0.008).

In addition, while the length of hospital stay is longer in the psychological distress group (64.74 days) compared to the group without distress (51.54 days), this difference is not statistically significant (*p* = 0.090). The results are similar to the days of stay in the ICU; there is a highly significant difference in the length of ICU stay between the two groups. COVID patients with psychological distress experience a substantially longer ICU stay (44.91 days) compared to those without distress (21.67 days) (*p* < 0.001).

A significant association is observed in the smoking status. In the distress group, 39.13% are smokers, whereas none are smokers in the no-distress group. Additionally, ex-smokers are more prevalent in the no-distress group (41.67%) compared to the distress group (17.39%) (*p* < 0.001).

There are notable differences in the prevalence of comorbidities: hypertension is more prevalent in the group without distress (58.33%) compared to the distress group (21.73%) (*p* = 0.004). Respiratory diseases are significantly more common in the distress group (43.75%) compared to the no-distress group (8.69%) (*p* = 0.003). Other comorbidities, including diabetes and cardiovascular diseases, do not show statistically significant differences between the two groups.

Differences in exercise capacity, physical function, and health status at hospital discharge are presented in Table 2.

The total SF-12 score was significantly higher in COVID-19 patients without psychological distress compared to COVID-19 patients with psychological distress (*p* < 0.001).

Results of the 6MWT have shown that while there is a numerical difference with patients with distress covering less distance (393.67 m) compared to those without distress (441.44 m), this difference is not statistically significant (*p* = 0.225).

The Borg post-test shows a significant difference between groups. Patients with distress report a higher Borg post-test score (6.44) compared to those without distress (2.07) (*p* < 0.001), indicating a greater perceived effort or difficulty during exercise.

The evaluation of physical function in the lower limbs reveals significant differences between the groups. Patients with distress have lower scores in both the right lower limb (52.66 vs. 75.15, *p* = 0.002) and the left lower limb (38.31 vs. 70.75, *p* < 0.001). These findings suggest impaired physical function in patients experiencing psychological distress.

Examining health status, the results have shown significant differences between both groups. Patients with distress have significantly lower scores in both the right upper limb (34.35 vs. 43.58, *p* = 0.048) and the left upper limb (28.60 vs. 39.90, *p* = 0.028).

In Table 3, the exercise capacity, physical function, and health status at 3 months after hospital discharge are presented.

At 3 months after hospital discharge, a statistically significant difference between the two groups in 6MWT is shown. Patients with psychological distress cover a shorter distance (374.10 m) compared to those without distress (468.85 m) (*p* = 0.002), indicating a potential limitation in exercise endurance for those experiencing psychological distress.

The Borg post-test also shows a significant difference between both groups in favor of the group without psychological distress. Patients with distress report a higher Borg post-test score (4.70) compared to those without distress (1.33) (*p* < 0.001), indicating a greater perceived effort or difficulty during exercise.

The evaluation of physical function in the lower limbs reveals significant differences between groups. Patients with psychological distress have lower scores in both the right lower limb (60.90 vs. 82.31, *p* = 0.001) and the left lower limb (62.38 vs. 77.91, *p* = 0.002).

Examining the health status, significant differences are noted between groups in favor of the psychological distress group. Patients with distress have lower scores in both the right upper limb (35.52 vs. 51.77, *p* < 0.001) and the left upper limb (35.53 vs. 47.94, *p* = 0.004).

Differences in exercise capacity, physical function, and health status at 6 months after hospital discharge are presented in Table 4.

At 6 months after hospital discharge, the 6MWT shows a statistically significant difference between the two groups. Patients with psychological distress cover a shorter distance (415.15 m) compared to those without distress (475.54 m) (*p* = 0.027).

The Borg post-test score also shows a significant difference between both groups in favor of the group without psychological distress. Patients with distress report a higher Borg post-test score (4.70) compared to those without distress (2.38) (*p* < 0.001), indicating a greater perceived effort or difficulty during exercise.

While the right lower limb’s physical function shows no statistically significant difference (*p* = 0.057), the left lower limb exhibits a significant difference. Patients with psychological distress have lower scores in the left lower limb (69.96 vs. 81.64, *p* = 0.027), suggesting potential impairment in physical function.

Finally, the assessment of the health status in the upper limbs does not reveal statistically significant differences between the two groups for both the right (*p* = 0.559) and left upper limbs (*p* = 0.234).

## 4. Discussion

This study aimed to determine whether post-hospitalization psychological distress is associated with exercise capacity, physical function, and health status in COVID-19 survivors.

Our results confirm the hypothesis of the study and have shown that COVID patients with psychological distress at hospital discharge reported a worse exercise capacity, physical function, and health status at hospital discharge, 3 months, and 6 months follow-up.

The prevalence of psychological distress in COVID patients at hospital discharge has been recently studied. Vlake et al. (2021) [38] reported that the prevalence of COVID patients with anxiety and depression one month after discharge was 25% and 26%, respectively, and remained at 3 months follow-up (17% and 22%, respectively). Nevertheless, to the best of our knowledge, this is the first attempt to study not only the prevalence, but also the relationship between psychological distress and exercise capacity, physical function, and health status.

Psychological distress and its repercussions have been studied in other respiratory diseases such as chronic obstructive pulmonary disease (COPD). Depression and anxiety have substantial repercussions in patients with COPD, contributing to a complex interplay that negatively impacts both physical and mental well-being. COPD patients experiencing depression and anxiety often face exacerbated symptoms, reduced adherence to treatment regimens, and impaired quality of life. The psychological distress can hinder pulmonary rehabilitation efforts, leading to decreased exercise tolerance and compromised pulmonary function. Additionally, the coexistence of depression and anxiety in COPD patients has been associated with increased healthcare utilization, higher rates of hospitalization, and elevated mortality risks. This bidirectional relationship between mental health and COPD is well-documented in scientific literature, with studies such as those by Atlantis and Fahey (2008) [39] and Yohannes et al. (2010) [40] highlighting the intricate connections and emphasizing the importance of integrated care strategies addressing both the respiratory and psychological aspects of COPD management. Effective interventions targeting depression and anxiety in COPD patients not only improve mental health outcomes, but also contribute to enhanced overall respiratory function and a better prognosis for these individuals.

In addition, Xu et al. (2008) [41] concluded that chronic obstructive pulmonary disease (COPD) patients with psychological problems had more hospitalizations and exacerbations compared to patients without psychological affectation. In the same line, Singh et al. (2016) [42] found that psychological disorders such as depression and anxiety contribute significantly to the 30-day readmission rate for patients with COPD. Previous research has concluded that psychological distress can weaken the immune system and increase vulnerability to respiratory infection in respiratory diseases [43,44].

Regarding the reported health status in subjects with psychological distress, Rodríguez-Torres et al. (2020) [37] evaluated the impact of psychological distress at hospital admission in respiratory disease patients. They evaluated 68 hospitalized patients with malignant pleural effusion and divided them into two groups according to their psychological distress. At discharge, patients with psychological distress obtained greater symptoms and a worse health status than those with lower psychological distress scores, even one month after hospitalization. In this line, our results in a similar sample size reveal an impact on physical function that lasts six months after hospitalization.

Our results have revealed that there were significantly more women in the groups with psychological distress. These results are in line with Gudmundsson et al. (2006) [45] who concluded that COPD women were more likely to suffer from more significant anxiety or depression. There could be more factors like more vulnerability to sociocultural factors associated with the mental impact, or more fluctuation in hormonal levels which are associated with emotional symptoms and make women more likely to report negative emotions than men [46,47].

The evaluation of psychological distress among patients holds significant implications across various domains. For patients themselves, identifying and addressing psychological distress can lead to improved mental well-being, potentially enhancing their overall quality of life and aiding in their recovery process. Managers within healthcare settings can utilize such evaluations to tailor support services and interventions, fostering a more patient-centered approach to care delivery. Hospitals stand to benefit by recognizing the importance of addressing psychological distress, as doing so can potentially reduce the length of hospital stays, readmission rates and healthcare costs. Societally, acknowledging and addressing psychological distress among patients can contribute to a more compassionate and empathetic healthcare system, promoting a culture of holistic patient care. From a broader healthcare perspective, integrating the evaluation of psychological distress into routine assessments can enhance the effectiveness of treatment plans, leading to better health outcomes and potentially reducing the burden on healthcare resources. Overall, evaluating psychological distress holds immense potential for positively impacting patients, healthcare providers, hospitals, society, and the healthcare system as a whole.

This study has some potential limitations. First, the lack of a structured psychiatric clinical interview or standardized measure of depression and anxiety. However, we based our study design on similar studies that have used the SF-12 to assess psychological distress [47,48]. Another limitation is that the study might have a limited sample size or lack diversity in its participant pool, which can affect the generalizability of the findings. In addition, this study cannot establish causation, only associations, which might limit the strength of the conclusions. Furthermore, more objective measures could have been used to avoid introducing biases or inaccuracies in the data. Finally, the absence of pre–COVID-19 pandemic assessment of enrolled patients means that we were not able to ascertain whether the psychological distress had been elicited by hospitalization or the pandemic. Potential confounding factors that could influence the results should have been taken into account.

Further studies should be conducted evaluating the effect of treatment of psychological distress, and also to analyze whether simple screening tests such as the SF-12 scale can be used to select those that could benefit from specific therapy based on future randomized trials. Furthermore, future studies with longer follow-up periods could be interesting to provide more comprehensive insights into the long-term effects of COVID-19.

## 5. Conclusions

COVID patients with psychological distress at hospital discharge reported worse exercise capacity, physical function, and health status at hospital discharge, 3 months, and 6 months follow-up. The presence of psychological distress is a factor to be taken into account in hospitalized patients with COVID-19. It has been related to symptom severity, exercise capacity, physical function and health status in the short and medium terms. The findings emphasize the need for integrated care approaches that address both the physical and psychological aspects of COVID-19 survivors, which can be significant for healthcare policy and practice.

## Figures and Tables

**Table 1:** Sociodemographic variables of the sample at hospital discharge.

Variable	COVID Patients with Psychological Distress Group (*n* = 23)	COVID Patients without Psychological Distress Group (*n* = 48)	*p*
Age (years)	56.87 ± 10.70	61.63 ± 12.70	0.126
Sex (% men)	78.26	95.83	0.020 *
BMI (kg/m^2^)	27.10 ± 3.33	30.93 ± 8.48	0.008 *
Length of hospital stay (days)	64.74 ± 28.55	51.54 ± 31.06	0.090
Length of ICU stay (days)	44.91 ± 25.85	21.67 ± 11.90	<0.001 **
Tabaquism			
Smokers (yes, %)	39.13	0	
Ex-smokers (yes, %)	17.39	41.67	<0.001 **
Non-smokers (yes, %)	43.47	58.34	
Comorbidities			
Hypertension (yes, %)	21.73	58.33	0.004 *
Diabetes (yes, %)	8.69	27.08	0.076
Cardiovascular diseases (yes, %)	17.39	18.75	0.890
Respiratory diseases (yes, %)	8.69	43.75	0.003 *

BMI: body mass index. ICU: intensive care unit. Variables are expressed as the mean ± standard deviation or percentage (%). * *p* < 0.05. ** *p* < 0.001.

**Table 2:** Differences between the groups in exercise capacity, physical function, and health status at hospital discharge.

Variable	COVID Patients with Psychological Distress Group (*n* = 23)	COVID Patients without Psychological Distress Group (*n* = 48)	*p*
SF-12 total score	55.29 ± 10.14	83.35 ± 16.65	<0.001 **
Exercise capacity			
6MWT (meters)	393.67 ± 73.51	441.44 ± 111.12	0.225
Borg post-test	6.44 ± 2.26	2.07 ± 1.62	<0.001 **
Physical function			
Right lower limb (Lb)	52.66 ± 21.21	75.15 ± 30.42	0.002 *
Left lower limb (Lb)	38.31 ± 17.36	70.75 ± 32.47	<0.001 **
Health status			
Right upper limb (Lb)	34.35 ± 13.54	43.58 ± 19.87	0.048 *
Left upper limb (Lb)	28.60 ± 13.57	39.90 ± 22.19	0.028 *

6MWT: 6 min walking test; Lb: pounds. Variables are expressed as the mean ± standard deviation. * *p* < 0.05. ** *p* < 0.001.

**Table 3:** Differences between the groups in exercise capacity, physical function, and health status at 3 months after hospital discharge.

Variable	COVID Patients with Psychological Distress Group (*n* = 23)	COVID Patients without Psychological Distress Group (*n* = 48)	*p*
SF-12 total score	61.47 ± 15.20	82.47 ± 15.40	<0.001 **
Exercise capacity			
6MWT (meters)	374.10 ± 119.97	468.85 ± 101.34	0.002 *
Borg post-test	4.70 ± 2.64	1.33 ± 1.92	<0.001 **
Physical function			
Right lower limb (Lb)	60.90 ± 17.19	82.31 ± 27.76	0.001 *
Left lower limb (Lb)	62.38 ± 11.34	77.91 ± 28.92	0.002 *
Health status			
Right upper limb (Lb)	35.52 ± 7.12	51.77 ± 20.27	<0.001 **
Left upper limb (Lb)	35.53 ± 11.05	47.94 ± 23.73	0.004 *

6MWT: 6 min walking test; Lb: pounds. Variables are expressed as the mean ± standard deviation. * *p* < 0.05. ** *p* < 0.001.

**Table 4:** Differences between the groups in exercise capacity, physical function, and health status at 6 months after hospital discharge.

Variable	COVID Patients with Psychological Distress Group (*n* = 23)	COVID Patients without Psychological Distress Group (*n* = 48)	*p*
SF-12 total score	70.02 ± 14.20	85.20 ± 12.23	<0.001 **
Exercise capacity			
6MWT (meters)	415.15 ± 80.94	475.54 ± 107.08	0.027 *
Borg post-test	4.70 ± 2.13	2.38 ± 2.23	<0.001 **
Physical function			
Right lower limb (Lb)	70.81 ± 20.06	82.27 ± 28.73	0.057
Left lower limb (Lb)	69.96 ± 15.28	81.64 ± 28.05	0.027 *
Health status			
Right upper limb (Lb)	64.27 ± 13.42	66.73 ± 21.46	0.559
Left upper limb (Lb)	58.56 ± 12.43	64.19 ± 27.07	0.234

6MWT: 6 min walking test; Lb: pounds. Variables are expressed as the mean ± standard deviation. * *p* < 0.05. ** *p* < 0.001.

## Data Availability

The analyzed data are available from the author upon reasonable request.

## References

[1] Wang C., Horby P.W., Hayden F.G., Gao G.F. (2020). A novel coronavirus outbreak of global health concern. Lancet.

[2] Zhu N., Zhang D., Wang W., Li X., Yang B., Song J., Zhao X., Huang B., Shi W., Lu R. (2020). A novel coronavirus from patients with pneumonia in China, 2019. N. Engl. J. Med..

[3] Statista COVID-26: Número Acumulado de Casos de Coronavirus (COVID-19) en el Mundo, Enero-Marzo [Internet]. Hamburgo: Statista; 26 de Enero de 2024. https://es.statista.com/estadisticas/1104227/numero-acumulado-de-casos-de-coronavirus-covid-19-en-el-mundo-enero-marzo/#:~:text=COVID%2D26%3A%20n%C3%BAmero%20acumulado%20de,en%20el%20mundo%202020%2D2023&text=A%20fecha%20de%202%20de,geograf%C3%ADa%20europea%20y%20del%20mundo.

[4] Zhao S.W., Li Y.M., Li Y.L., Su C. (2023). Liver injury in COVID-19: Clinical features, potential mechanisms, risk factors and clinical treatments. World J. Gastroenterol..

[5] Wang B., Yu Y., Yu Y., Wang N., Chen F., Jiang B., Chen Y., Zhang J., Liu J., Wang H. (2023). Clinical features and outcomes of hospitalized patients with COVID-19 during the Omicron wave in Shanghai, China. J. Infect..

[6] Conti V., Corbi G., Sabbatino F., De Pascale D., Sellitto C., Stefanelli B., Bertini N., De Simone M., Liguori L., Di Paola I. (2023). Long COVID: Clinical framing, biomarkers, and therapeutic approaches. J. Pers. Med..

[7] Lavrentieva A., Kaimakamis E., Voutsas V., Bitzani M. (2023). An observational study on factors associated with ICU mortality in Covid-19 patients and critical review of the literature. Sci. Rep..

[8] Tola H.H., Shojaeizadeh D., Garmaroudi G., Tol A., Yekaninejad M.S., Ejeta L.T., Kebede A., Karimi M., Kassa D. (2015). Psychological distress and its effect on tuberculosis treatment outcomes in Ethiopia. Glob. Health Action.

[9] Spencer-Segal J.L., Smith C.A., Slavin A., Sampang L., DiGiovine D., Spencer A.E., Zhang Q., Horowitz J., Vaughn V.M. (2021). Mental health outcomes after hospitalization with or without COVID-19. Gen. Hosp. Psychiatry.

[10] Mazza M.G., De Lorenzo R., Conte C., Poletti S., Vai B., Bollettini I., Melloni E.M.T., Furlan R., Ciceri F., Rovere-Querini P. (2020). Anxiety and depression in COVID-19 survivors: Role of inflammatory and clinical predictors. Brain Behav. Immun..

[11] Gutub A., Shambour M.K., Abu-Hashem M.A. (2023). Coronavirus impact on human feelings during 2021 Hajj season via deep learning critical Twitter analysis. J. Eng. Res..

[12] Huang H.C., Huang L.K., Hu C.J., Chang C.H., Lee H.C., Chi N.F., Shyu M.L., Chang H.J. (2014). The mediating effect of psychological distress on functional dependence in stroke patients. J. Clin. Nurs..

[13] Sorel J.C., Veltman E.S., Honig A., Poolman R.W. (2019). The influence of preoperative psychological distress on pain and function after total knee arthroplasty: A systematic review and meta-analysis. Bone Joint J..

[14] Linares-Moya M., Rodríguez-Torres J., Heredia-Ciuró A., Granados-Santiago M., López-López L., Quero-Valenzuela F. (2022). Psychological distress prior to surgery is related to symptom burden and health status in lung cancer survivors. Support. Care Cancer.

[15] Smith A.B., Jones A.D., Serlachius A.S., Johnson D.W. (2023). The impact of acute psychological stress on the immune system: A systematic review. PLoS ONE.

[16] Johnson C., Smith L., Anderson M. (2020). Psychological stress and medication adherence in hospitalized patients. J. Health Psychol..

[17] Ware J., Kosinski M., Keller S.D. (1996). A 12-Item Short-Form Health Survey: Construction of scales and preliminary tests of reliability and validity. Med. Care.

[18] Vilagut G., Valderas J.M., Ferrer M., Garin O., López-García E., Alonso J. (2008). Interpretación de los cuestionarios de salud SF-36 y SF-12 en España: Componentes físico y mental [Interpretation of SF-36 and SF-12 questionnaires in Spain: Physical and mental components]. Med. Clin..

[19] Ware J.E., Kosinski M., Turner-Bowker D.M., Gandek B. (2002). How to Score Version 2 of the SF-12 Health Survey (With a Supplement Documenting Version 1).

[20] Gill S.C., Butterworth P., Rodgers B., Mackinnon A. (2007). Validity of the mental health component scale of the 12-item Short-Form Health Survey (MCS-12) as measure of common mental disorders in the general population. Psychiatry Res..

[21] Fleishman J.A., Selim A.J., Kazis L.E. (2010). Deriving SF-12v2 Physical and Mental Health Summary Scores: A Comparison of Different Scoring Algorithms. Qual Life Res..

[22] Borg G.A. (1982). Psychophysical bases of perceived exertion. Med. Sci. Sports Exerc..

[23] American Thoracic Society (2002). ATS statement: Guidelines for the six-minute walk test. Am. J. Respir. Crit. Care Med..

[24] Solway S., Brooks D., Lacasse Y., Thomas S. (2001). A qualitative systematic overview of the measurement properties of functional walk tests used in the cardiorespiratory domain. Chest.

[25] Macchiavelli A., Giffone A., Ferrarello F., Paci M. (2021). Reliability of the six-minute walk test in individuals with stroke: Systematic review and meta-analysis. Neurol. Sci..

[26] Enright P.L. (2003). The six-minute walk test. Respir. Care.

[27] Singh S.J., Morgan M.D., Scott S., Walters D., Hardman A.E. (1992). Development of a shuttle walking test of disability in patients with chronic airways obstruction. Thorax.

[28] Ingle L., Rigby A.S., Nabb S., Jones P.K., Clark A.L., Cleland J.G. (2014). Clinical determinants of poor six-minute walk test performance in patients with left ventricular systolic dysfunction and no major structural heart disease. Eur. J. Heart Fail..

[29] Walker P.P., Burnett A., Flavahan P.W., Calverley P.M. (2008). Lower limb activity and its determinants in COPD. Thorax.

[30] Bohannon R.W., Andrews A.W. (1987). Interrater reliability of hand-held dynamometry. Phys. Ther..

[31] Mentiplay B.F., Perraton L.G., Bower K.J., Adair B., Pua Y.H., Williams G.P., McGaw R., Clark R.A. (2015). Assessment of lower limb muscle strength and power using hand-held and fixed dynamometry: A reliability and validity study. PLoS ONE.

[32] Shephard R.J., Montelpare W., Plyley M., McCracken D., Goode R.C. (1991). Handgrip dynamometry, Cybex measurements and lean mass as markers of the ageing of muscle function. Br. J. Sports Med..

[33] Taekema D.G., Gussekloo J., Maier A.B., Westendorp R.G., de Craen A.J. (2010). Handgrip strength as a predictor of functional, psychological and social health. A prospective population-based study among the oldest old. Age Ageing.

[34] Bohannon R.W. (2008). Hand-grip dynamometry predicts future outcomes in aging adults. J. Geriatr. Phys. Ther..

[35] Leong D.P., Teo K.K., Rangarajan S., Lopez-Jaramillo P., Avezum A., Orlandini A., Seron P., Ahmed S.H., Rosengren A., Kelishadi R. (2015). Prognostic value of grip strength: Findings from the Prospective Urban Rural Epidemiology (PURE) study. Lancet.

[36] Rodríguez Torres J., Cabrera Martos I., López López L., Torres Sánchez I., Granados Santiago M., Valenza M.C. (2020). Psychological distress at hospital admission is related to symptoms severity and health status in malignant pleural effusion patients. Eur. J. Cancer Care.

[37] Vlake J.H., Wesselius S., van Genderen M.E., van Bommel J., Boxma-de Klerk B., Wils E.J. (2021). Psychological distress and health-related quality of life in patients after hospitalization during the COVID-19 pandemic: A single-center, observational study. PLoS ONE.

[38] Atlantis E., Fahey P. (2008). Cochrane Review: Bidirectional association between depression and chronic disease among women. J. Am. Med. Womens Assoc..

[39] Yohannes A.M., Willgoss T.G., Baldwin R.C., Connolly M.J. (2010). Depression and anxiety in chronic heart failure and chronic obstructive pulmonary disease: Prevalence, relevance, clinical implications and management principles. Int. J. Geriatr. Psychiatry.

[40] Xu W., Collet J.P., Shapiro S., Lin Y., Yang T., Platt R.W., Wang C., Bourbeau J. (2008). Independent effect of depression and anxiety on chronic obstructive pulmonary disease exacerbations and hospitalizations. Am. J. Respir. Crit. Care Med..

[41] Singh G., Zhang W., Kuo Y.F., Sharma G. (2016). Association of Psychological Disorders With 30-Day Readmission Rates in Patients With COPD. Chest.

[42] Herbert T.B., Cohen S. (1993). Depression and immunity: A meta-analytic review. Psychol. Bull..

[43] Irwin M.R., Miller A.H. (2007). Depressive disorders and immunity: 20 years of progress and discovery. Brain Behav. Immun..

[44] Gudmundsson G., Gislason T., Janson C., Lindberg E., Suppli Ulrik C., Brøndum E. (2006). Depression, anxiety and health status after hospitalisation for COPD: A multicentre study in the Nordic countries. Respir. Med..

[45] Pagel M.D., Erdly W.W., Becker J. (1987). Social networks: We get by with (and in spite of) a little help from our friends. J. Pers. Soc. Psychol..

[46] Hambisa S., Siraj J., Mesafint G., Yimam M. (2021). Assessment of Psychological Distress and Associated Factors among Hospitalized Patients During the COVID-19 Pandemic at Selected Hospitals in Southwest Ethiopia. Neuropsychiatr. Dis. Treat..

[47] Saks B.R., Glein R.M., Jimenez A.E., Ankem H.K., Sabetian P.W., Maldonado D.R., Lall A.C., Domb B.G. (2022). Patients Obtain Meaningful Clinical Benefit After Hip Arthroscopy Despite Preoperative Psychological Distress: A Propensity-Matched Analysis of Mid-Term Outcomes. Arthroscopy.

[48] Prapa P., Papathanasiou I.V., Bakalis V., Malli F., Papagiannis D., Fradelos E.C. (2021). Quality of Life and Psychological Distress of Lung Cancer Patients Undergoing Chemotherapy. World J. Oncol..

